# Photosynthetic Responses of *Racomitrium japonicum* L. to Strontium Stress Evaluated through Chlorophyll a Fluorescence OJIP Transient Analysis

**DOI:** 10.3390/plants13050591

**Published:** 2024-02-22

**Authors:** Hui Ren, Yunmei Lu, Yunlai Tang, Peng Ren, Hao Tang, Qunlong Chen, Peigang Kuang, Renhua Huang, Wenkun Zhu, Ke Chen

**Affiliations:** 1 School of Life Science and Engineering, Southwest University of Science and Technology, Mianyang 621010, China; renhui@mails.swust.edu.cn (H.R.); tyl@swust.edu.cn (Y.T.); renpeng@swust.edu.cn (P.R.); zhuwenkun@swust.edu.cn (W.Z.); 2 College of Biological Engineering, Jingchu University of Technology, Jingmen 448000, China; lym79616@163.com; 3 Ecological Protection and Development Research Institute of Aba Tibetan and Qiang Autonomous Prefecture, Aba 623000, China; edwindon6668@163.com; 4 Administration Bureau of Jiuzhaigou National Nature Reserve, Jiuzhaigou 623402, China; qunlong_chen@163.com (Q.C.); peigang_kuang@163.com (P.K.); 5 Engineering Research Center of Biomass Materials, Ministry of Education, Southwest University of Science and Technology, Mianyang 621010, China

**Keywords:** Sr^2+^ stress, moss, electron transport, chlorophyll a fluorescence kinetics, JIP test

## Abstract

Nuclides pollution and its biological effects are of great concern, especially for bryophytes during their terrestrial adaptation. Understanding PSII activity and electron transport response is vital for comprehending moss abiotic stress reactions. However, little is known about the photosynthetic performance of moss under nuclide treatment. Therefore, this study aimed to evaluate the chlorophyll fluorescence of *Racomitrium japonicum* L. The moss was subjected to Sr^2+^ solutions at concentrations of 5, 50, and 500 mg/L to evaluate chlorophyll *a* fluorescence using the OJIP test. Moderate and high Sr^2+^ stress led to inner cell membrane dissolution and reduced chlorophyll content, indicating impaired light energy absorption. At 5 mg/L Sr^2+^, fluorescence kinetics showed increased light energy capture, energy dissipation, and total photosynthetic driving force, thus stimulating transient photosynthetic activity of PSII and improving PSI reduction. Linear electron transfer and PSII stability significantly decreased under moderate and high Sr^2+^ stress, indicating potential photosynthetic center damage. Cyclic electron transfer (CEF) alleviated photosynthetic stress at 5 mg/L Sr^2+^. Thus, low Sr^2+^ levels stimulated CEF, adjusting energy flux and partitioning to protect the photosynthetic apparatus. Nevertheless, significant damage occurred due to inefficient protection under high Sr^2+^ stress.

## 1. Introduction

Environmental factors such as light, water, and heavy metals play a crucial role in influencing plant growth. Stress can arise from either the absence or excess of these factors [1,2]. Among the cellular functions, chloroplast photosynthetic activity is the most sensitive function. Therefore, investigating plant photosynthesis under stressful conditions is an important research avenue [3,4]. Recently, it has been observed that when plants grow in soils with excessive metal ion content, they experience a significant inhibition of O_2_ evolution, CO_2_ fixation, and phosphorylation capabilities [5].

High temperatures affect photosynthetic membranes, leading to grana stacking loss owing to the dissociation of the peripheral antenna complex of photosystem II (PSII) from its core complex. This results in reduced energy connectivity within the unit and destabilization of the PSII structure, posing a threat to overall PSII photosynthetic activity [4,6,7]. In addition to temperature and pressure, the deactivation of photoreaction centers is associated with environmental metal ion toxicity. Studies on the photosynthetic performance of *Chlorella pyrenoidosa* have shown that high copper (Cu) concentrations significantly inhibit photosynthesis and respiration. The absorption flux (ABS/RC) at each PSII reaction center increases with increasing Cu concentration. In contrast, the electron transport flux (ET0/RC) decreases. The decline in efficiency, with which a trapped exciton can move an electron into the electron transport chain further than QA^−^ (Ψ_0_), the maximal quantum yield of primary photochemistry (ϕP_0_), and the quantum yield of electron transport (ϕE_0_) were also observed [8]. These findings highlight the significant impact of abiotic environmental factors on electron transport during photosynthesis and various enzyme systems [9]. Similar to heavy metal pressure, radioactive strontium (90 Sr) is released into the environment through reactions such as nuclear fission [10,11], causing environmental pollution after settling in the form of atmospheric, water, and soil cycles [12]. According to a survey, compared with the strontium concentration in nature (160 mg/g), the soil Sr reached 247 mg kg to form a stable pollution [13,14]. The content of strontium in the soil is therefore typically higher than that of Ni, Cu, or Zn [15], and a large amount of Sr accumulation has been observed in some plants exposed to water culture, such as cabbage, lettuce, etc. [16]. Accumulation of millimolar amounts of stable Sr induces chemotoxic effects and damage in several plants; it affects plant’s development, photosynthesis, metabolism, and genetics, especially photosynthetic factors, which are very sensitive to environmental changes, so environmental pollutant strontium (Sr) also influences photosynthetic responses [12,17,18].

Among the earliest plant types, Bryophytes exhibit more sensitivity and resilience in their response to environmental changes and survival under adverse conditions [19]. Hence, exploring the photosynthetic response of bryophytes to Sr is highly significant as a fluorescent probe for environmental changes.

Over the past two decades, in vivo fast chlorophyll a fluorescence rise kinetics (OJIP) and JIP test analysis have been extensively employed to monitor alterations in the photosynthetic apparatus under different stress conditions. This technique is susceptible to environmental changes, offering nondestructive, precise, and rapid characteristics [20,21,22]. It is a potent tool effectively employed to assess photosynthesis in various plants via non-invasive methods [23]. Photosynthetic materials containing oxygen exhibit a fluorescence uptick within the 1st second of illumination after each stress treatment, followed by a distinctive fluorescence increase in the shape of the OJIP curve [24]. The rapid chlorophyll fluorescence rise kinetics typically exhibits a sequence of phases from the initial (FO) to the maximal (FM) fluorescence value, labeled step O (20 µs), J (≈2 ms), I (≈30 ms), and P (≈300 ms) [25]. These stages align with the redox states of photosystems PSII and PSI, reflecting the efficiency of electron transfer through the system to the terminal electron acceptor on the PSI acceptor side [26].

Besides the basic OJIP steps, additional phases may emerge under specific conditions, such as the L-step (reflecting the energetic connectivity of the PSII units), K-step (associated with the inactivation of the oxygen-evolving complex, OEC), or H- and G-steps observed in corals and foraminifers [22,27]. However, the positions of these steps are not permanently fixed in the typical curve. They might shift toward shorter or longer times in response to different stress conditions [28]. Several researchers have consistently highlighted that measuring OJIP transients serves as a sensitive and reliable method for detecting and quantifying environmental changes in PSII and PSI of plants [29,30]. We performed a widely used chlorophyll a (Chl a) fluorescence kinetic analysis on dark-adapted leaf samples in numerous data comparisons. Various parameters were calculated to characterize the steady state of the photosynthetic apparatus, thereby validating the reliability of the fluorescence analysis [31]. For example, alterations in OJIP transients induced by high temperatures have been employed to assess heat tolerance in common bean cultivars, apple leaves, and peels [32,33]. Furthermore, the diverse effects of chromate on the photosynthetic organs of *Spirulina* can also be evaluated by OJIP transients [34]. However, in bryophytes, it is unclear whether the OJIP transient can be used as a stress indicator and how photosynthetic physiological responses change under nuclide stress. Therefore, this study aimed to evaluate the chlorophyll a fluorescence of *R. japonicum* L.

## 2. Results

### 2.1. Effects of Sr^2+^ on Cell and Chlorophyll a Contents in R. japonicum

**Figure 1:**
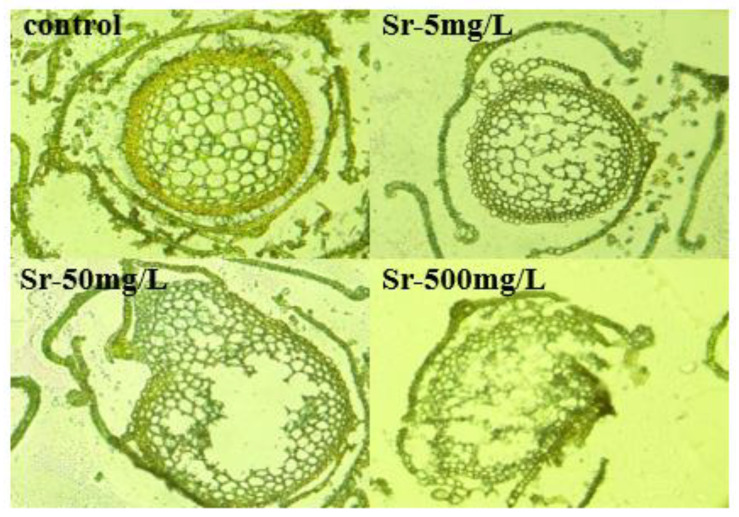
Cell morphology in *R. japonicum* measured at different strontium concentrations (0, 5, 50, 500 mg/L).

The moss phenotypes exhibited variability after treatment with three strontium concentrations. To investigate these effects at the cellular level, we employed frozen section technology to observe moss stem tips using a 20× optical fluorescence microscope. Figure 1 illustrates the progressive deterioration of moss cell lumens with increasing strontium concentration. At 500 mg/L strontium, considerable damage occurred, characterized by blurred cell borders and abnormal inclusions. Conversely, a concentration of 5 mg/L Sr^2+^ resulted in minimal damage, with no observed broken cells, which was not significantly different from that of the control. Therefore, high Sr concentrations are believed to cause plant cell damage. Photosynthesis—a susceptible physiological process in plants—responds to environmental stresses, such as light, temperature, and water. To further investigate the photosynthetic performance of plants in response to injury, we measured changes in chlorophyll content. Table 1 shows that the chlorophyll a content initially increased and then declined with increasing concentrations, signifying the inhibition of chlorophyll a and b synthesis under medium-to-high strontium concentrations. At a Sr^2+^ concentration of 5 mg/L, the chlorophyll a content increased by 6.9%, which was not significantly different from that of the control. However, at 50 and 500 mg/L, a significant decrease of approximately 30% was observed. Among cellular functions, chloroplast photosynthetic activity stands out as highly sensitive, with chlorophyll a playing a significant role in absorbing and capturing light energy within chloroplasts, which is crucial for photosynthesis. Therefore, we can conclude that strontium does affect the photosynthetic performance of mosses.

### 2.2. Effects of Sr^2+^ on Chl a Fluorescence Rise Kinetics in R. japonicum

#### 2.2.1. Raw Fluorescence Rise Kinetic OJIP Curves and Relative Variable Fluorescence *V_t_*

**Figure 2:**
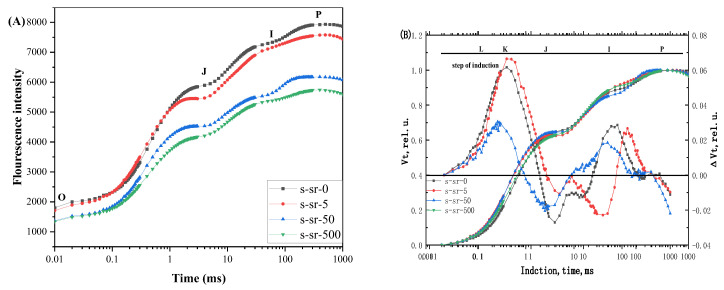
Chl a fluorescence rise kinetics of *R. japonicum* at different strontium concentrations (0, 5, 50, 500 mg/L). (**A**) shows it is normalized by *F_O_* and *F_M_* as *V_t_* = (*F_t_* − *F_O_*)/(*F_M_* − *F_O_*) and Δ*V_t_* = *V_t_* (treatment) − *V_t_* (control) in a logarithmic time scale. (**B**) shows the original kinetics. Each value of point in curve represents the average of 20 measurements.

The rapid chlorophyll a fluorescence rise kinetic OJIP and JIP test analysis, based on the “Theory of Energy Fluxes in Biomembranes”, have proven to be a potent and widely utilized tool for investigating plant growth status. Its nondestructive, accurate, and rapid characteristics, and its sensitivity to environmental changes such as high temperature, drought, heavy metals, and salt stress make it highly effective [35]. Figure 2A depicts the fluorescence rise kinetic OJIP curves of *R. japonicum* treated with 5, 50, and 500 mg/L or 0 mg/L (control) of strontium. Different Sr^2+^ treatments exhibited varying impacts on the fluorescence increase kinetics of the OJIP curve compared with the control. The J-step level increased with the 5 mg/L Sr^2+^ solution, indicating increased sensitivity. With the concentration increasing from 0 to 500 mg/L, the J-step gradually approached F_P_, leading to the disappearance of the IP phase of the OJIP curve. The fluorescence rise in moss maintained a complete OJIP multiphase transient curve during the 500 mg/L treatment, suggesting that strontium treatment at this stage did not completely inhibit photosynthetic activity. Additionally, a significant increase was observed in the *F_O_* value (20 μs), affecting the *F_M_* value with the Sr^2+^ levels rising, leading to a noticeable decrease in the *F_O_/F_M_.* The change in *F_O_* value is related to the modification of the structure and order of the light-harvesting complexes during solar energy absorption. A decline in *F_O_* suggests a gradual degradation of antenna pigments, leading to reduced absorption of light energy from the environment. The kinetics of fluorescence increase in the leaves were suggested to differ significantly from those of the control group at low and medium Sr^2+^ levels. However, with the increase in treatment concentration, the characteristics of the fluorescence rise kinetics in the OJIP curves post-strontium treatment varied with different concentrations, indicating that nuclides change in fluorescence rise kinetics.

To further investigate the effect of strontium stress on the fluorescence rise kinetic OJIP curves, we double-normalized the fluorescence curves between *F_O_* (20 μs) and *F_M_*, and the curves were presented as relative variable fluorescence *V_t_* = (*F_t_* − *F_O_*)/(*F_M_* − *F_O_*) and Δ*V_t_* = *V_t_* (treatment) − *V_t_* (control) at logarithmic time scale to show invisible features of Chl a fluorescence rise kinetic OJIP curve (Figure 2B). The *V_t_* and ∆*V_t_* results showed the emergence of two distinct phases in the fluorescence rising kinetic curve: the L-step and the K-step. The primary alteration in the fluorescence increase kinetics of *R. japonicum* was the appearance of positive K and I peaks, dependent on Sr^2+^ concentration. The fluorescence rise kinetic OJIP of the moss underwent a gradual transformation into an OKJIP pattern, introducing a new intermediate “K” step at approximately 300 µS. Along with the induction of the K-step, a sharp decrease occurred in the variable chlorophyll fluorescence intensity and *p*-value under 500 mg/L strontium stress. This suggests the possibility of an I-step (reflecting the energetic connectivity of PSII units), a K-step (related to the inactivation of the OEC), or H-steps and G-steps in corals and foraminifers [35]. There was a clear correlation between the fluorescence intensity of OJIP, changes in fluorescence kinetics, and variations in strontium concentration.

#### 2.2.2. The L-Band

**Figure 3:**
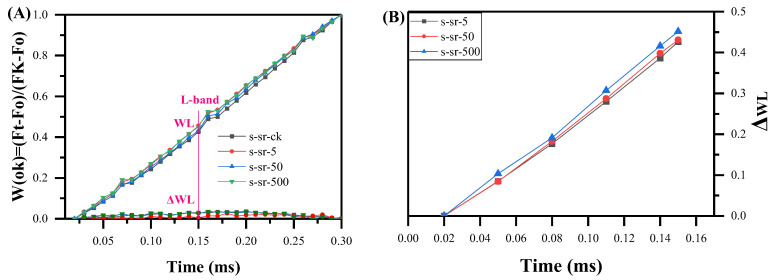
Fluorescence rise kinetics normalized by *F_O_* and *F_K_* as *W_OK_* = (*F_t_* − *F_O_*)/(*F_K_* − *F_O_*), (**A**) and the value of Δ*W_L_* as Δ*W_L_* = (*F*_150µs_ − *F_O_*)/(*F_J_* − *F_O_*), (**B**) in a linear time scale from 0 to 300 μs of *R. japonicum* at different strontium concentrations (0, 5, 50, 500 mg/L). Each curve represents the average of 20 measurements.

To reveal more details within the fluorescence rise kinetic curves and assess events in the OK, OJ, OI, and IP phases, we conducted additional normalizations and corresponding curve subtractions using the principle of differential kinetics. In Figure 3A, fluorescence rise kinetic curves from various treatments have been double-normalized by O (20 μs) and K-steps (300 μs) to reveal a weak L-band over the exponential period of 0–300 μs. By employing the kinetics *W_OK_* = (*F_t_* − *F_O_*)/(*F_K_* − *F_O_*), we extracted the unique fluorescence characteristics of the L-band, hidden between O and K, which was obtained at approximately 150 μs after data processing. The L-band serves as an indicator of the energetic connectivity or grouping of PSII units and is more prominent when the connectivity or grouping probability is lower [35]. It was observed that the 500 mg/L Sr^2+^ treatment increased the L-band. In contrast, the other three concentrations exhibited a slight increase in the L-band. Across the four concentration treatments, the L-band indicators positively correlated with concentration, revealing a significant concentration-dependent decline in energy connectivity based on the positive L-band. Additionally, the L-band can be quantified further through the relative fluorescence of *F_J_* − *F_O_*, denoted as Δ*W_L_* = (*F*_150µs_ − *F_O_*)/(*F_J_* − *F_O_*) [36]. Δ*W_L_* values showed differences after the three different strontium concentration treatments, with overlapping ∆*W_L_* curves for low and moderate Sr^2+^ levels but a significant increase in the L-band with higher Sr^2+^ levels. Thus, moss exhibited reduced PSII energy connectivity at strontium concentrations exceeding 500 mg/L. Lower connectivity leads to decreased excitation energy utilization and diminished PSII unit stability.

#### 2.2.3. The K-Band

**Figure 4:**
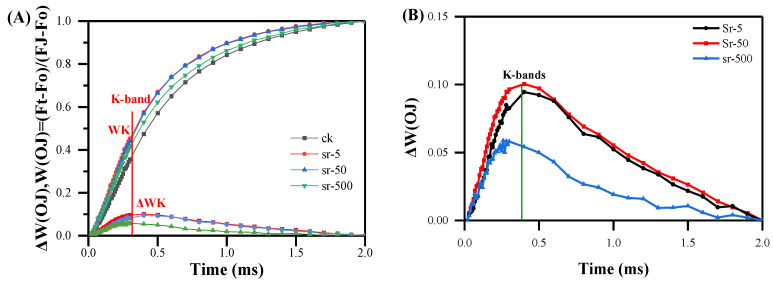
Fluorescence rise kinetics normalized by *F_O_* and *F_J_* as *W_OJ_* = (*F_t_* − *F_O_*)/(*F_J_* − *F_O_*), (**A**) and the difference kinetics Δ*W_OJ_* = *W_OJ_* (treatment) − *W_OJ_* (control), (**B**) in a linear time scale from 0 to 2 ms of *R. japonicum* at different strontium concentrations (0, 5, 50, 500 mg/L). Each curve represents the average of 20 measurements.

In Figure 2B, the presence of K-step has been identified. To assess the effects of various treatments on the K-step, Chl a fluorescence rise kinetics were normalized by O-step (20 μs) and J-step (2 ms), presented as *W_OJ_* = (*F_t_* − *F_O_*)/(*F_J_* − *F_O_*) and Δ*W_OJ_* = *W_OJ_* (treatment) − *W_OJ_* (control) in the linear time scale from 0–2 ms (Figure 4A). Under normal conditions, the K phase did not appear. While the four peaks were not consistently well separated, a distinct representation of Δ*W_OJ_* was executed to confirm that the K- and J-steps represent distinct phenomena and elucidate differences in the K phase among treated concentrations (Figure 4B). Δ*W_OJ_* curves indicated that the three strontium concentrations induced the occurrence of the K-bands, with a more pronounced positive K-band evident in the different kinetic ∆*W_OJ_* with increasing concentrations. In particular, under the 50 mg/L strontium treatment, the intensity of the change in the K phase was more active and evident than that of the 5500 mg/L strontium treatment. We hypothesize that plants were more responsive to this concentration, experiencing enhanced damage, resulting in a very high Chl a fluorescence intensity, mirroring the trend observed in Figure 2B. Furthermore, upon scrutinizing the variations in phases, we observed that the positions of the phases are not consistently fixed and they may shift to shorter or longer times under varying stress conditions. The duration of the K phase was prolonged, reaching the K phase at about 400 μs instead of 300 μs. An increase in the K-step suggests the inactivation of OEC centers at the PSII donor side, amplifying the inhibition of linear electron transfer as the accumulation of electrons in the P_680_^+^ center increases. This implies that strontium treatment likely induced oxidative damage in *R. japonicum*. Regarding the reduction in the K phase following the 500 mg/L strontium concentration, it could be inferred that, besides severely impaired linear electron transport, oxidative damage in moss might have inhibited the water cleavage system, intensifying PSI damage and consequently diminishing the photosynthetic response of *R. japonicum*.

#### 2.2.4. The IP Phase

**Figure 5:**
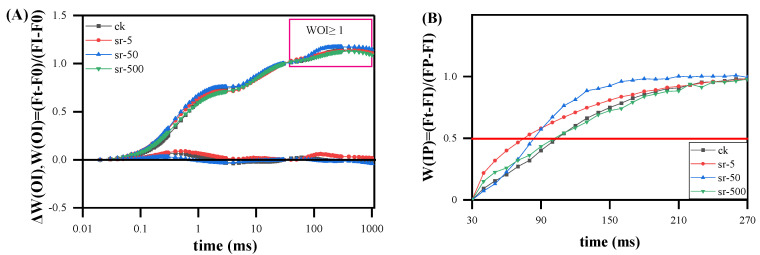
Fluorescence rise kinetics normalized by *F_O_* and *F_I_* as *W_OI_* = (*F_t_* − *F_O_*)/(*F_I_* − *F_O_*), the difference kinetics Δ*W_OI_* = *W_OI_* (treatment) − *W_OI_* (control) (**A**) and normalized by *F_I_* and *F_P_* (*F_M_*) as *W_IP_* = (*F_t_* − *F_I_*)/(*F_P_* − *F_I_)*; in a logarithmic time scale of *R. japonicum* at different strontium concentrations (0, 5, 50, 500 mg/L). *W_IP_* = 0.5 is the half-time of the rising curves (**B**). Each curve represents the average of 20 measurements.

Fluorescence kinetic curves were normalized between the O-step and I-step (30 ms) using *W_OI_* = (*F_t_* − *F_O_*)/(*F_I_* − *F_O_*) and Δ*W_OI_* = *W_OI_* (treatment) − *W_OI_* (control) on a logarithmic time scale (Figure 5A). Δ*W_OI_* was employed to show the effects of the four concentrations on the J-step, aligning with the findings of Δ*V_t_*. The maximum amplitude of the fluorescence rise (*W_OI_* ≥ 1) for each *W_OI_* curve in the linear time range of 0.02–300 ms reflects the size of the pool of the end electron acceptors at the PSI acceptor side [37]. Comparatively, concerning the control (0 mg/L), moss amplitude was slightly reduced at high concentrations (500 mg/L), and the *W_OI_* amplitude showed no significant increase between the 5 and 50 mg/L Sr^2+^ treatments. The amplitude strength was linked to the inhibition of pool size, and none of the three concentrations resulted in significant changes in the *W_OI_* amplitude. Moreover, the J and K phases are not consistently synchronous; they manifest as two independent steps in the increase in the kinetics of chlorophyll fluorescence. Strontium treatment may not significantly excite the J phase. Moreover, the electron pool size is independently regulated regardless of the change in its reduction rate. The change in the reduction rate of the electron acceptor pool at the end of the PSI acceptor side after different sample treatments can be explored using another fluorescence increase kinetically normalized to *W_IP_* (normalized with I- and P-steps). In Figure 5B, the IP phase illustrated for *W_OI_* ≥ 1 plotted on a linear time scale from 30 ms to 270 ms was calculated as *W_IP_* = (*F_t_* − *F_I_*)/(*F_P_* − *F_I_*). *W_IP_* represents the reduction rate of the end electron acceptor in PSI, with a lower (or higher) conductivity generally mirrored in a larger (or lower) half-life. Therefore, the time point at *W_IP_* = 0.5 (the half-time of the rising curves) can be employed to reflect the reduction rate of the PSI end electron acceptor pool [38]. The half-rise-time values of the treated moss samples surpassed those of the control (approximately 100 ms), while those of the 500 mg/L strontium-treated samples (approximately 90 ms) were lower than those of 50 mg/L (approximately 75 ms) and 5 mg/L (approximately 60 ms). This suggests that the Sr concentration treatment increased the reduction rates of the end electron acceptors on PSI, with low Sr^2+^ levels of terminal electron acceptors at the highest reduction rate on PSI, followed by middle concentrations, and the lowest reduction rate at high Sr^2+^ levels. The low and middle concentrations of strontium were more sensitive to the stimulation of the photosynthetic system of moss, intensifying the PSI reaction center activity. Consequently, this acceleration of electron reduction ensured the fluidity of the linear electron transfer pathway, thereby promoting moss activity to some extent.

### 2.3. The Energy Fluxes in R. japonicum

**Figure 6:**
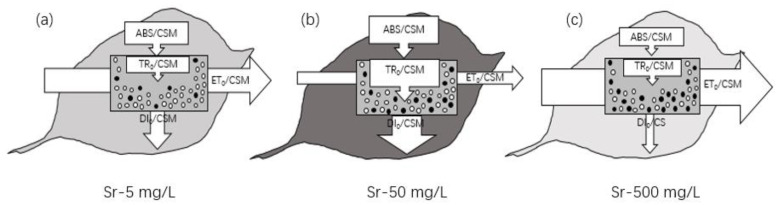
Comparison of PSII behavior at various strontium concentrations. The PSII behavior illustrates the phenomenological energy fluxes (per excited cross-section, denoted as CSM) in the leaf model (**a**–**c**). The width of each arrow corresponds to the value of the representative energy flux, with reaction centers per cross-section (RC/CSM) represented by small circles. The white circles represent active reaction centers per cross-section (RC/CSM), while the black cir-cles represent inactivated ones.The darkness of the leaf area, indicating chlorophyll density, provides additional visualization of the ABS/CSM flux.

Different concentrations of strontium can be effectively assessed for their impact on energy fluxes using the energy flow model of the cell, employing the energy pipeline models of the photosynthetic apparatus [39,40,41]. This dynamic model shows the fluctuation in the value of each energy flux over time. Each energy flux value, influenced by different concentrations of Sr treatment—or different environmental conditions in general—is expressed by the appropriately adjusted width of the corresponding arrow. Figure 6 presents the pipeline models for mosses treated with 5, 50, and 500 mg/L Sr solutions. The pipeline model has two types of expressions: one refers to the reaction center in the membrane, dealing with the specific energy fluxes (per reaction center, RC), while the other concerns the excited cross-section of a leaf, focusing on phenomenological energy fluxes (per excited cross-section, here taken as CSM) [42]. In this experiment, we chose the latter expression to ensure a more uniform energy transfer. In the leaf model, active reaction centers per cross-section (RC/CSM) are indicated by small empty circles, while inactivated ones are represented by solid black circles. The darkness of the leaf area, indicating chlorophyll density, provides additional visualization of the ABS/CSM flux. The leaf model demonstrated that applying different Sr concentrations resulted in (Figure 6a–c): (a) A 5% decrease in ABS/RC at 5 and 500 mg/L, with an increase of 13.5% at 50 mg/L. ABS captures solar energy through the antenna, and excited chlorophyll molecules are reduced at both extreme concentrations (5 and 500 mg/L). (b) A slight (<10%) decrease in TR_0_/CSM at both 5 and 500 mg/L, while there was a 13% increase at 50 mg/L, indicating that trapped energy is conducted to the reaction center TR, and the rate of reduction involved in QA subsequently increases through the medium electron transfer complex. (c) A more pronounced decrease (>10%) in the ET_0_/CSM; in the case of the 5 and 500 mg/L Sr^2+^ treatments, the decrease in the ET_0_/CSM was only 3% and 0.6%, respectively. A strontium concentration of 50 mg/L stimulated the electron reduction rate, but electron transfer (ET) decreased substantially. (d) A substantial (>10%) increase in DI_0_/CSM; the decrease was reduced to 7% and 12% at low and high Sr^2+^ levels, respectively. Electrons are partly used for CO_2_ fixation in the photosystem, consuming a certain amount of energy. Conversely, with the transfer of electrons, most energy that cannot be used is dissipated through heat and fluorescence. Moreover, the ability to dissipate is enhanced with a substantial increase in the DI_O_ ratio at 50 mg/L. Figure 6 illustrates the effect of strontium on the distribution and transfer of light energy, especially the 50 mg/L strontium concentration treatment absorbed, transferred, and transmitted a large amount of energy, much of which was dissipated. Combined with the previous fluorescence performance at low and high concentrations, moss must have experienced damage to energy transfer while affecting electron transfer under the stress treatment at high concentrations.

### 2.4. JIP-Test Analysis in R. japonicum

**Figure 7:**
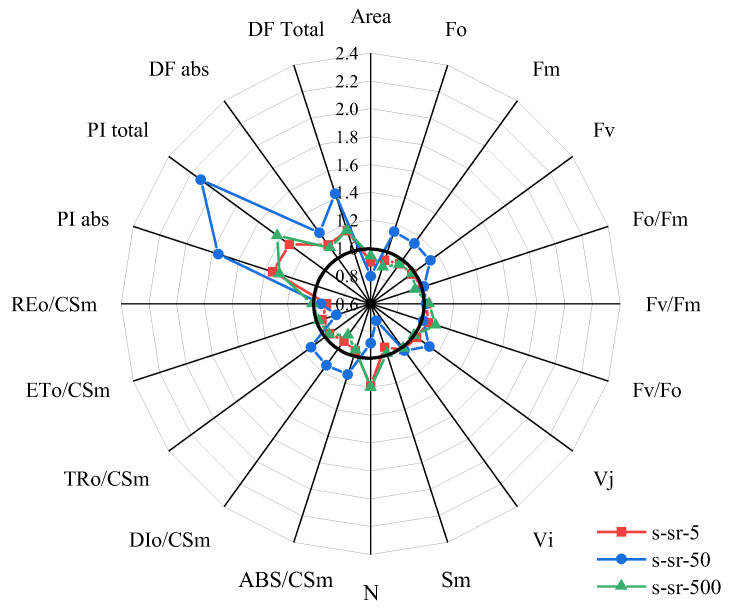
Spider plot presenting several JIP-test parameters quantifying PSII structure and function of the photosynthetic apparatus in *R. japonicum* at 5, 50, 500 mg/L strontium concentrations. Each parameter is expressed as a fraction relative to 0 mg/L values.

Both OEC impairment and a contrast in photosynthetic fluxes characterize the sensitivity of photosynthetic fluorescence to 50 mg/L strontium treatment. To further assess the effect of different strontium concentrations on photosynthesis, we assessed several JIP test parameters that quantify the conformation, structure, and function of the photosynthetic apparatus [43]. In Figure 7, moss treated with a 500 mg/L Sr^2+^ solution showed a slight decrease in *F_O_*, *F_M_*, *F_J_*, and *F_I_* compared to the control, with *F_O_* significantly reduced by 12%. While these four parameters exhibited a significant increase, the 50 mg/L treatment demonstrated a more pronounced effect. The corresponding ratios *F_O_/F_M_*, *F_V_/F_M_*, and *F_V_/F_O_* displayed a decreasing trend at high concentrations and an increase at low and medium concentrations, but with no significant effect observed for the different concentration treatments. This suggests a reduction in both variable and actual fluorescence following high-concentration treatment, leading to changes in the maximum quantum yield of primary photochemistry and impairment of PSII activity. Moreover, the 50 mg/L concentration stimulated the reaction efficiency of PSII, resulting in a significant increase in the overall photosynthetic activity with ABS/CSM, TR_0_/CSM, and DI_0_/CSM increasing by approximately 15%, aligning with the findings in Figure 6. Plants exhibited sensitivity and were more active in capturing and absorbing fluorescence after the intermediate Sr^2+^ concentration treatment. In addition to the thermal dissipation, which consumes part of the absorbed and trapped large amounts of electrons, the reduction in transferred electrons (ET_0_/CSM) also contributed to a weakening of the PSII linear electron transfer rate. The increase in photosystem activity and the substantial absorption of light energy must be consumed through other electron transfer pathways, affecting performance indices (PI) and DF. Regarding energy conservation efficiency, the performance index (PI) of PSII exhibited a substantial increase at different strontium concentrations, susceptible at 50 mg/L, where PI_ABS_ and PI_total_ increased by 29% and 43%, respectively. As an indicator of the activity of each intercellular function, moss PI values significantly rose with strontium treatment at every concentration. This suggests that strontium treatment stimulated photosynthesis, with the intensity of the total photosynthetic drive, the driving force of absorbed light (DF_ABS_), and the overall driving force between photosystems (DF_total_) increasing by 10% and 16% at 50 mg/L compared to the control, surpassing the increments observed in the other two treatments. At 500 mg/L, Sr treatment stimulated PSII energy conservation efficiency, exhibiting a pronounced increase in overall PSII photosynthetic activity. Analyzing all the performance indicators in Figure 7, the Sr concentration level correlated with photosynthetic activity. It caused damage to the assembled structure of PSII pigments while stimulating concurrently photosynthetic activity. The somewhat positive impact on PSII activity, Chl concentration, and the assembled structure of PSII pigments at an Sr^2+^ concentration of 50 mg/L was particularly noteworthy. However, the adverse effect observed on linear electron transfer, impeding electron transfer from PSI to PSII, suggests the existence of an alternative electron transfer pathway for electrons accumulated through antenna pigment absorption.

### 2.5. Photosynthetic Electron Transport in R. japonicum

**Figure 8:**
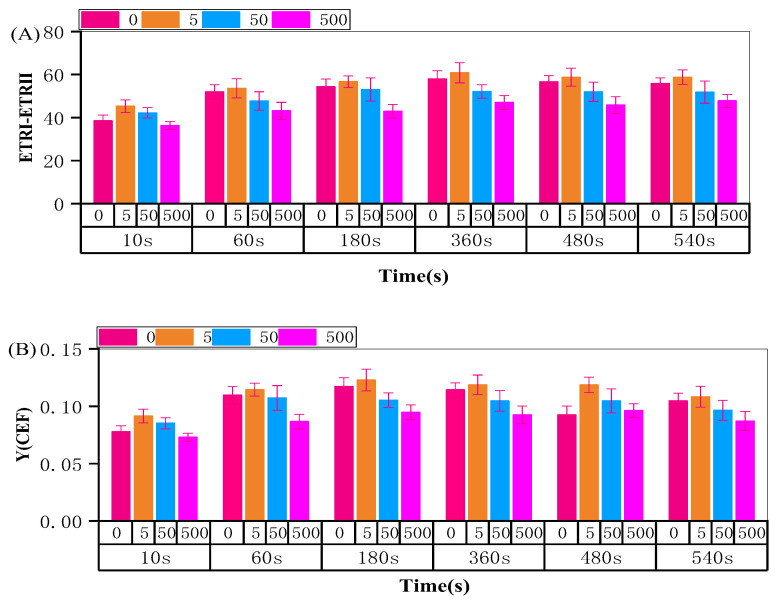
The rate (ETRI–ETRII) (**A**) and size (YCEF) of electron transfer, (**B**) in *R. japonicum* at 0, 5, 50, 500 mg/L strontium concentration. Data are the mean ± SD (*n* = 4).

Under normal circumstances, electron transfer in plants typically follows a linear pattern. However, exposure to stress prompts the involvement of cyclic electrons in the transfer process [44]. To assess the sensitivity of photosynthetic activity to 50 mg/L Sr treatment, we calculated the cycle electrons of the PSI reaction center using a Dual-PAM 100 measuring system (Heinz Walz, Effeltrich, Germany) by subtracting ETRII_120s_ from ETRI_120s_. As shown in Figure 8A, with increased concentration intensity, ETRI–ETRII rapidly increased in the first 10 s and stabilized after 1 min. The initial increase in ETRI–ETRII was primarily attributed to Sr^2+^ concentration stimulation, while the subsequent stable state resulted from the regulation of electron transport pathways. The same trend applied to the CEF capacity, calculated as Y (I)–Y (II). Compared with the control group, an increase in concentration to 50 and 500 mg/L led to a significant decrease in CEF. However, the time to reach equilibrium shortened, and excitation occurred at low levels (Figure 8B). These findings suggest that cyclic electron transfer accelerates with middle-to-high Sr^2+^ levels, and the electron pool size and translocation ability can be stimulated at lower levels. Integrating the observations from each phase of Chl a fluorescence rise kinetics and JIP-test analysis, we found that the electron transfer of the photosystem was under a certain pressure in the presence of middle and high Sr^2+^ concentrations in moss. The capacity of PSI to receive electrons decreases and the electron transfer rate slows, significantly impeding the normal linear electron transfer. This pressure tended to induce cyclic electrons in the middle- and high-concentration solutions, exhibiting a transient increase before stabilizing. Further in-depth studies are required to elucidate the specific response mechanism for the excitation of cyclic electrons in mosses.

## 3. Discussion

### 3.1. Chl a Fluorescence Rise Kinetic Response of Moss under Sr^2+^ Stress

Photosynthesis is responsive to alterations in the plant environment [45,46]. Plants exhibit a stress response to nucleophiles similar to that of most heavy metals [47,48]. However, there is limited understanding of the response of chlorophyll fluorescence in mosses to nuclide treatment. Chl a fluorescence rise kinetics and the JIP test are susceptible and informative tools extensively utilized to monitor changes in the photosynthetic apparatus under various stress conditions [37]. Our experiments revealed that strontium significantly affected the photosynthetic fluorescence of moss. All OJIP phases exhibited variability, with increasing strontium concentrations progressively inhibiting chlorophyll fluorescence and a more pronounced K-step (OKJIP) [49]. The K-step is associated with the activity of the OEC on the donor side of PSII. Extensive studies have established that the K-peak occurs due to the disruption of the oxidation system, leading to a significant reduction in the continuous electron supply from the OEC to the RC and PSII receptors. During linear electron transfer from QA to QB, the reoxidation of QA^−^ to QA prompts a short-lived rise in the reduced Pheo-/QA-concentration, resulting in a transient increase in the activity of the reaction center, resulting in a K-peak maximum [50,51]. The mosses exhibited a K-peak, reaching their maximum value after a strontium concentration of 50 mg/L, representing an approximately 20% increase compared to treatments with low and high concentrations, positively stimulating their PSII activity. The dip observed after the K-step could be attributed to the deficiency of electrons from the donor side. PSII RCs are reopened by electron transfer from Q_A_^−^ to Q_B_, eventually accumulating P680+ centers with a low fluorescence yield [52]. While each plant may perceive environmental factors such as light, heat, and water differently, the simultaneous occurrence of the L-band and K-band, causing a rapid rise in fluorescence in moss leaves under strontium-stressed conditions, holds a better predictive value for plants that have experienced oxidative damage [35,53]. We observed that the elevated strontium concentration leads to the appearance of L-band and K-band, which appear with a certain delay (Figure 2 and Figure 3). We presumed that strontium exerts pressure on the moss during this delay period. Further investigations are required to explore the pressure range induced by different metals. Besides photoactivity, the L-band indicators are associated with photosynthesis and energy dynamics. At medium-to-high concentrations, the L-band indicators rise, resulting in poor utilization of energy connectivity in photosynthesis, significantly affecting the energy synergy between PSII units [22].

### 3.2. Photosynthetic Organ Response of Mosses under Sr^2+^ Stress

In addition to the observed K-steps and L-steps, ABS/CSM and TR_0_/CSM further demonstrate that strontium significantly affects PSII RCs. When the moss was exposed to a concentration of 50 mg/L Sr^2+^, the activity of PSII reaction centers behaved differently from other concentrations; not only did it elevate the specific rate of exciton capture by RCs, but the transient boost in photosynthetic activity following strontium stimulation also accelerated energy dissipation in the unit reaction center (DI_0_/CSM). This process helped in consuming the excess accumulation of electrons in the reaction center, achieving a photoprotective effect. At this point, the energy captured per reaction center for Q_A_ reduction (TR_0_/CSM) increased. In contrast, linear electron transfer (ET_0_/CSM) was hindered. However, no irreversible impairment of photosynthetic activity was observed, even at a concentration of 500 mg/L Sr^2+^. Hence, there appears to be an alternative transfer pathway for significant amounts of reduced electrons, ensuring the continued activity of the photosystem reaction center. Furthermore, a concentration of 50 mg/L acted as a turning point, leading to an exceptionally active behavior in the photosynthetic performance index PI_ABS_, with the same trend observed in the total photosynthetic driving force (DF). This aligns with findings by Strasser et al., who argue that PI_ABS_ serves as a sensitive criterion for identifying different stress sensitivities, surpassing the sensitivity of conventional *F_V_*/*F_M_* [22]. Based on the previous analysis, we hypothesize that the significant increase in the overall photosynthetic activity of PSII (PI_ABS_) is attributed to the stimulation of RCs (RC/CS) by strontium.

### 3.3. Electron Transport Response of Moss under Sr^2+^ Stress

Contrary to a study on temperature stress by Havaux, where temperature-induced heat affected the photosystem, stimulating PSII but inhibiting PSII activity in pea leaves [54], our investigation revealed that at a Sr^2+^ concentration of 50 mg/L, the rising IP phase and the time of *W_IP_* (*t* = 0.5) were significantly shorter. This indicates an increase in the pool size and an acceleration in the reduction rate of terminal electron acceptors in PSI. Compared to low and middle Sr^2+^ levels, the reduction rate on the PSI receptor side decreased at 500 mg/L Sr^2+^. The maximal amplitude of *W_OI_* ≥ 1 reflects the size of the pool of the end electron acceptors at the PSI acceptor side, while the WIP represents the reduction rate of the end electron acceptor in PSI [37,38]. This suggests that high concentrations of Sr mainly inhibit PSII electron flow beyond *Q_A_*. Through the analysis of each OJIP phase, it is clear that the linear electron transfer pathway is inhibited, and the IP phase corresponds to the reduction of PC^+^ and P_700_^+^ [55,56]. To ensure an increased rate of electron reduction on the PSI receptor side at 50 mg/L, the accumulated electrons could only pass through cyclic or pseudo-cyclic electron transfer pathways. Under the same conditions, our moss electron response test indicated that higher concentrations more readily excited the size and capacity of the electron transfer cycle. This is consistent with the results of Xia and Ren’s study in moss [57,58]. Further studies are required to determine the specific response of different concentrations to the cyclic electron transfer pathway.

## 4. Materials and Methods

### 4.1. Plant Materials and Treatments

In this study, we used the moss *R. japonicum* collected from Zhangjiajie Forest Park, Hunan Province, China. After thoroughly rinsing with deionized water, the moss was subjected to experiments to remove gravel and weeds. Subsequently, *R. japonicum* was cultivated in a greenhouse with moderate relative humidity (70–80%), day/night temperatures (23/17 °C), and 30% full sunlight for 2 days before undergoing 88Sr^2+^ treatment. Treatment solutions of Sr^2+^ were prepared using 88SrCl_2_·6H_2_O. In this study, there were four treatments including control (0 mg/L Sr^2+^), Sr-5 (5 mg/L Sr^2+^), Sr-50 (50 mg/L Sr^2+^), and Sr-500 (500 mg/L Sr^2+^). Each treatment includes four replications with 10 g of moss per replicate tray. Plants were treated in a plastic tray for 7 days, with daily 2-h soaking at noon. All three treatments and the controls (no treatment) were arranged in a completely randomized design, with each treatment using five samples from each of the four trays for that treatment. After 7 days of greenhouse cultivation, we measured the cell structure, chlorophyll content, Chl a fluorescence rise kinetic curve, and chlorophyll fluorescence parameters of the plants. The data obtained during the determination of Chl a fluorescence rise kinetic curve were the average value of 20 measurements, because the instant light exposure of each measurement produces high quantum strong light stimulation, causing temporary damage to plants. Each sample was about 0.5 g, a total of 20 samples, and JIP tests were conducted successively to improve the reliability of the data.

### 4.2. Cell Structure Observation and Chlorophyll Content Determination

One week after Sr^2+^ treatment, the plants were surface-rinsed with deionized water. Subsequently, the same parts, approximately 3 cm in length after removing the rhizoids, were cut and positioned in an embedding box under the fixation of tweezers. SAKURA4583 embedding solution was added to the box, ensuring complete solidification of the plants in the freezer at −40 °C. The embedded block was then sliced to a thickness of 5 μm using a frozen slicer and affixed onto a slide. The structure and morphology of the cells were observed using a 20× fluorescence optical microscope. Fresh leaves (0.3 g) were finely ground, and chlorophyll content was measured. Chlorophyll content determination was conducted through spectrophotometry in the dark, as described by Gitelson [59]. The physiological index was repeated three times, and the average value was calculated.

### 4.3. Chl a Fluorescence Kinetic Measurement and JIP Test

**Figure 9:**
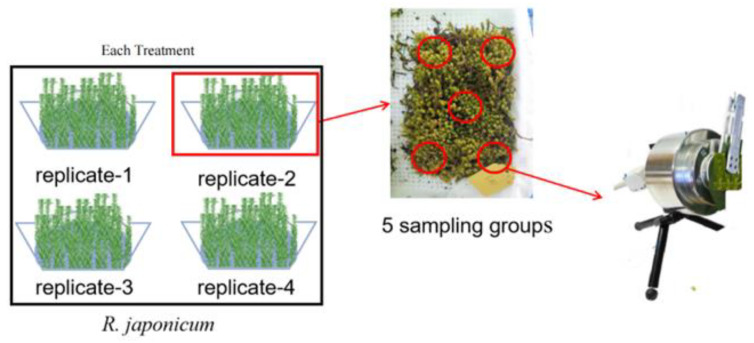
Setup diagram for chlorophyll fluorescence kinetic measurement.

The setup diagram for chlorophyll fluorescence kinetic measurement was shown in Figure 9. Chl a fluorescence rise kinetic OJIP curves were determined using 1 s pulses of continuous red light (3000 μmol (photons) m^−2^ s^−1^) with MPEA fluorometer (Hansatech Instruments Ltd., King’s Lynn, UK) as described by Strasser and Govindjee [24]. Fluorescence data were recorded with a variable sampling rate, ranging from 0.01 to 0.3 ms, with data recorded every 10 μs. The article referred to several fundamental sets of data: *F_O_* (the fluorescence intensity at 20 μs), *F_L_* (the fluorescence intensity at 150 μs), *F_K_* (the fluorescence intensity at 300 μs), *F_J_* (the fluorescence intensity at 2 ms), *F_I_* (the fluorescence intensity at 30 ms), and the maximal fluorescence intensity *F_M_* is equal to *F_P_*.

Chlorophyll fluorescence values in the OJIP part were computed through double normalization within distinct time intervals of the transient—OP, OK, OJ, OI, and IP: *V_t_*, representing relative variable fluorescence at time *t*, was calculated using the formula *V_t_* = (*F_t_* − *F_O_*)/(*F_M_* − *F_O_*); *W_OK_*, indicating the ratio of variable fluorescence *F_t_* − *F_O_* to the amplitude *F_K_* − *F_O_*, served to clarify L-band; *W_OJ_*, calculated by *W_OJ_* = (*F_t_* − *F_O_*)/(*F_J_* − *F_O_*), was employed to show K-band; *W_OI_*, determined through the formula *W_OI_* = (*F_t_* − *F_O_*)/(*F_I_* − *F_O_*), was commonly utilized to show J-step; *W_IP_*, with formula *W_IP_* = (*F_t_* − *F_I_*)/(*F_P_* − *F_I_*), was associated with electron flow through PSI and inactive ferredoxin-NADP+-reductase (FNR) at the acceptor side of PSI [56,60].

The data were analyzed according to the JIP test [22,25]. The JIP test establishes the maximal (subscript “o”) energy fluxes in the energy cascade for the following events: absorption (ABS), trapping (TR_0_), electron transport (ET_0_), dissipation (DI_0_), reduction of end acceptors of PSI (RE_0_), and excited leaf cross-section (CS).

The maximum quantum yield of PSII primary photochemistry, Po, is expressed as TR_0_/ABS = 1 − (*F_O_*/*F_M_*). The probability that an electron progresses beyond QA^−^ is defined as Eo = ET_0_/TR_0_ = (1 − VJ). The quantum yield for the reduction of the end electron acceptors at the PSI acceptor side (Ro) is given as Ro = (1 − VI)/(1 − VJ). To convey the overall photosynthetic activity of PSII, two indices were introduced: the performance index on an absorption basis, PI (PI_ABS_/PI_TOTAL_) and DF (DF_ABS_/DF_total_).

$${PI}_{ABS}=\frac{RC}{ABS}\times \left(\frac{\varphi_{P_{0}}}{1-\varphi_{P_{0}}}\right)\times \left(\frac{\psi_{E_{0}}}{1-\psi_{E_{0}}}\right)$$

$${PI}_{total}={PI}_{ABS}\times \left(\frac{\delta_{R_{0}}}{1-\delta_{R_{0}}}\right)$$

$${DF}_{ABS}=\log{(PI}_{ABS})$$

$${DF}_{total}=\log{(PI}_{total})$$

The photosynthetic electron transfer rate was computed using the following equation: ETRI = PPFD × Y (I) × 0. 84 × 0.5; ETRII = PPFD × Y (II) × 0.84 × 0.5, where ETRI and ETRII represent the electron transfer rates through PSI and PSII, respectively, ETRI–ETRII represents the cyclic electron transfer (CEF) rates, and PPFD represents the photosynthetic effective radiation intensity.

### 4.4. Data Analysis

One-way analysis of variance (ANOVA) was carried out and means were separated with Duncan’s LSD at 95% using SPSS Statistics 26.0. All figures were generated using the OriginLab software (version 9.0).

## Figures and Tables

**Table 1:** Chlorophyll content in *R. japonicum* measured at varying strontium concentrations.

Treatment (Sr^2+^ mg/L)	Chl (mg/g)	Chl a (mg/g)	Chl b (mg/g)
0	101.00 ± 1.06 a	69.68 ± 0.96 a	31.33 ± 0.10 a
5	106.51 ± 0.35 a	74.50 ± 0.23 a	32.01 ± 0.13 a
50	67.50 ± 2.47 b	46.35 ± 2.12 b	21.15 ± 0.35 b
500	66.10 ± 3.57 b	47.07 ± 2.97 b	19.03 ± 0.62 b

Note: Significant differences denoted by letters (a and b) at *p* < 0.05 exist between Sr treatment group in moss (LSD test). Data are the mean ± SD (*n* = 4).

## Data Availability

Data available on request from the corresponding author. The data are not publicly available due to privacy.
